# Modulation of APC Function and Anti-Tumor Immunity by Anti-Cancer Drugs

**DOI:** 10.3389/fimmu.2015.00501

**Published:** 2015-09-29

**Authors:** Kea Martin, Jens Schreiner, Alfred Zippelius

**Affiliations:** ^1^Department of Biomedicine, University Hospital Basel, University of Basel, Basel, Switzerland; ^2^Department of Medical Oncology, University Hospital Basel, Basel, Switzerland

**Keywords:** chemotherapy, dendritic cells, anti-tumor immunity, antigen-presenting cell, tumor-induced T cell dysfunction, immunogenic cell death

## Abstract

Professional antigen-presenting cells (APCs), such as dendritic cells (DCs), are central to the initiation and regulation of anti-cancer immunity. However, in the immunosuppressive environment within a tumor APCs may antagonize anti-tumor immunity by inducing regulatory T cells (Tregs) or anergy of effector T cells due to lack of efficient costimulation. Hence, in an optimal setting, anti-cancer drugs have the power to reduce tumor size and thereby may induce the release of tumor antigens and, at the same time, modulate APC function toward efficient priming of antigen-specific effector T cells. Selected cytotoxic agents may revert APC dysfunction either by directly maturing DCs or through induction of immunogenic tumor cell death. Furthermore, specific cytotoxic agents may support adaptive immunity by selectively depleting regulatory subsets, such as Tregs or myeloid-derived suppressor cells. Perspectively, this will allow developing effective combination strategies with novel immunotherapies to exert complementary pressure on tumors via direct toxicity as well as immune activation. We, here, review our current knowledge on the capacity of anti-cancer drugs to modulate APC functions to promote durable anti-cancer immune responses.

## DCs are Central to the Initiation and Regulation of Anti-Cancer Immunity

Both the induction of endogenous anti-tumor immune responses and the successful implementation of immunotherapy protocols rely on adequate activation of adaptive immunity by antigen-presenting cells (APCs). Although innate and adaptive immune cells act in concert to fight cancer cells, T cells play a superior role in inducing and maintaining sustained anti-tumor immune responses (1–4). Professional APCs include B-cells, macrophages, dendritic cells (DCs), and skin-resident Langerhans cells. Among these, DCs are by far the most potent activators of adaptive immunity owing to their unique capacity to induce primary T cell responses (5, 6).

To initiate T cell responses in the tumor setting, DCs must first recognize tumor cells as “abnormal” cells. Tumor cells differ from normal cells due to expression of altered-self or neo-antigens that arise as a consequence of genetic instability and high mutation rates in transformed cells (7, 8). Efficient activation of naïve and central memory T cells requires at least three signals delivered by APCs (9, 10). Along with the uptake of tumor-associated antigens and presentation in the context of MHC molecules, professional APCs are further required to provide lymphocyte costimulation, such as through expression of the B7 molecules (CD80 and CD86) or CD40. Expression of T cell-directing cytokines and additional costimulatory surface receptors by DCs subsequently provides the impulse for appropriate CD4^+^ T helper cell polarization (11). Importantly, DC maturation is regulated by the type, duration, and timing of danger/stress signals, such as pathogen-associated molecular patterns (PAMPs) or damage-associated molecular patterns (DAMPs), both of which trigger DC-intrinsic pattern-recognition receptors (12–14). Consequently, these signals determine the quality and quantity of costimulation provided by DCs and thus have the power to define the outcome of T cell immunity.

Tumor cells may undergo cell death due to hypoxia and nutrient deprivation (15) resulting in the release of host-derived DAMPs as an indicator of a dying or “stressed” cell. Thus, on theoretical grounds, tumors may provide the basic elements needed for the initiation of successful anti-tumor immune responses. However, it is well established that the immune-suppressive microenvironment of progressive tumors may severely interfere with both APC and T cell activation, thereby limiting the induction of endogenous anti-tumor immunity and the success of immunotherapies. Importantly, numerous tumor-mediated mechanisms may induce DC dysfunction, leading to immature or semi-mature DCs that are incapable of providing the necessary activation signals (16, 17). Consequently, under these circumstances, antigen presentation will typically provoke T cell tolerance including T cell anergy, peripheral T cell clonal deletion, or the induction of regulatory T cells (Tregs) (18–21).

In light of the recent success of novel immunotherapy approaches, future therapeutic efforts will ultimately focus on the development of effective combination strategies that exert complementary pressure on tumors via immune activation and additional direct toxicity. Of note, accumulating evidence reveals unrecognized immune-modulatory features of chemo- and radiotherapy (22, 23). In addition to reducing the primary tumor burden and thereby, at least in part, reverting the immunosuppressive microenvironment, specific compound classes can also induce DC maturation, enhance antigen cross-presentation, selectively eliminate immunosuppressive cells, or induce immunogenic cell death (ICD) (24, 25). These mechanisms may provide the basis for initiation of anti-tumor immunity and therefore support the successful implementation of T cell-mediated immunotherapy protocols, such as blockade of inhibitory receptors. Consequently, chemo-immunotherapy protocols are being evaluated in numerous clinical studies with promising therapeutic activity (26).

A major drawback of cytotoxic anti-cancer agents is their possible interference with T cell activation and clonal expansion (27, 28). A precise definition of the immune-modulating properties of cytotoxic therapies is, therefore, crucial for optimizing chemo-immunotherapy regimens. Here, we review the current knowledge on the capacity of anti-cancer drugs to modulate the phenotype and function of APCs, and in particular, the impact of these agents on T cell effector functions.

## Modulation of APC Function by Anti-Cancer Chemotherapeutics

### Direct DC maturation by cytotoxic anti-cancer agents

A lack of T cell effector function is mostly caused by inefficient expression of costimulatory molecules on tumor-associated DCs or by dysregulation of DC maturation pathways (29). Therefore, understanding how cytotoxic agents influence DC maturation is vital for designing effective chemo-immunotherapy protocols. An improved DC phenotype associated with T cell activation *in vitro* was reported after treatment of immature DCs with the topoisomerase I inhibitor topotecan. In contrast, treatment of lipopolysaccharide-matured DCs with topotecan resulted in decreased allogeneic T cell responses accompanied with a shift toward T_H_2 responses and increased IL-10 in co-cultures (30). Therefore, topoisomerase inhibitors might down-modulate responses of previously activated tumor-resident DCs, suggesting limited suitability for T cell immunotherapy combinations. Studies on other topoisomerase I inhibitors report conflicting results on DC maturation (31, 32). Also, using a DC-based reporter system, nine of 12 investigated compounds were identified as DC stimulatory (33). The effects of topoisomerase inhibitors on the induction of anti-tumor immunity as well as T cell activation and expansion *in vivo*, therefore, require further investigations.

Liu and colleagues revealed mechanistic insights into the molecular events associated with chemotherapy-induced DC maturation (32). Expression of the cell cycle regulator p21^waf1/cip1^ in human DCs was associated with a favorable DC phenotype and was shown to be upregulated by cytotoxic agents. p21^waf1/cip1^ expression correlated with enhanced expression of CD83 and CD86 in response to the anti-malaria agent artesunate and several anti-cancer compounds including camptothecin, lenalidomide, and docetaxel (32). When considering the “two-signal model” in innate immune activation (34), it seems plausible that p21, which generally indicates intrinsic cell stress, is activated in response to cytotoxicity. This “cell stress” or “abnormal condition” in the innate cell itself may deliver the necessary secondary signal for complete activation of innate immune cells and therefore result in enhanced DC maturation.

Along the same line, microtubule-destabilizing agents (MDAs), such as the *Vinca*-alkaloids, dolastatins, or maytansines, can directly affect DC maturation. Early studies indicated that microtubule disruption by colchicine, vinblastine, and vincristine induced marked expression of IL-1 in monocytes (35). Interestingly, rupture of the actin filaments by cytochalasins could not recapitulate this effect. Broad immune-stimulatory effects on murine DCs upon colchicine or vinblastine treatment, including expression of further pro-inflammatory cytokines and enhanced cross-presentation, have subsequently been confirmed by Takashima and colleagues (33, 36, 37). In extension of those data, we were able to demonstrate that two further families of MDAs, the dolastatins and maytansines, potently induced DC maturation. Importantly, DC pre-treatment with these agents induced profound T cell immunity, while treatment of tumor-bearing mice synergized with blockade of CTLA-4 and PD-1. Mechanistically, tumor rejection could be explained by enhanced infiltration of lymphocytes into the tumors and a shift toward an increased effector T cell to Treg ratio (38–40). Experiments to elucidate DC signaling pathways induced by MDAs are currently ongoing.

In stark contrast, we did not detect significant changes in DC ­phenotype or cytokine expression upon exposure to microtubule-­stabilizing agents (MSA), such as the taxane-family of compounds (38). Various studies have reported modulation of DC phenotype upon exposure to low, non-cytotoxic, concentrations of paclitaxel and other chemotherapeutics (41–44). However, these studies mostly evaluated DC function and phenotypic maturation in the context of paclitaxel pre-treated tumor cells or in combination with lipopolysaccharide treatment. Direct effects of paclitaxel and other MSAs on DCs were generally very moderate and thus are largely consistent with our data. The stimulatory effects of paclitaxel on tumor-associated macrophages, which subsequently may lead to activation of intra-tumoral immune cells, such as DCs, NK cells, and cytotoxic T lymphocytes, are comprehensively reviewed elsewhere (45).

Antibody-drug conjugates (ADCs) are emerging as powerful treatment strategies with outstanding target specificity and high therapeutic activity in cancer patients. The immune-modulatory capacities of dolastatins and maytansines are of particular clinical interest as their synthetic analogs, i.e., monomethyl auristatin E (MMAE) and DM1, are used as cytotoxic payloads of ADCs (46). Importantly, in tumor-bearing mice, DC activation upon treatment with such ADCs is equally potent as observed after administration of the respective free compound. Upon internalization, the cytotoxic payload is released into the tumor cell cytoplasm but may also diffuse into the surrounding microenvironment (47). Notably, the latter may induce maturation of tumor-resident DCs. We detected increased CD8 and CD4 T cell infiltrates, activation of APCs and T cells as well as reduced Treg frequencies in patients treated with the MMAE-carrying ADC Brentuximab Vedotin (BV) (39). Furthermore, induction of long-lasting tumor-specific T cells was detected in relapsed lymphoma patients responding to BV (with or without donor lymphocyte infusions) post-allogeneic HSCT (48, 49).

### DC stimulation via immunogenic cell death

Apoptotic cell death was historically considered to be non-immunogenic. However, some types of cell death have been demonstrated to induce an immune response against antigens released from dying cells, commonly referred to as immunogenic cell death (ICD). Immunogenic signals released by dying tumor cells can induce antigen uptake as well as antigen processing and presentation by the APC. Although cytotoxic anti-cancer therapies generally induce apoptosis, ICD is only induced in treatment with some of these agents, particularly, anthracyclines (50), oxaliplatin (51), and cyclophosphamide (52) as well as for irradiation (50). ICD is characterized by the induction of endoplasmic reticulum (ER) stress and autophagy, which is in distinction to non-immunogenic apoptosis (53, 54). Hallmarks of ICD include the pre-apoptotic exposure of calreticulin (CRT) on the cell surface, the secretion of adenosine triphophosphate (ATP), and the post-apoptotic release of the chromatin-binding protein high-mobility group box 1 (HMGB1) (50, 55, 56). Importantly, the suppression of each of these signals abolishes the immunogenicity of cell death, demonstrating the non-redundancy of each of these pathways (50, 55, 56).

Cytotoxic agents that trigger ICD are also efficient inducers of CRT cell-surface exposure. CRT is under normal circumstances located at the membrane of the ER. Following the induction of an ER stress response, CRT translocates to the cell surface where it serves as an established “eat me signal” for apoptotic cells (50, 54). This occurs well before the induction of apoptotic changes, such as the surface exposure of phosphatidylserine. Binding of CRT to CD91 on phagocytes induces phagocytosis and macropinocytosis leading to the efficient clearance of these cells (57). CRT is also detectable on the surface of viable cells; however, the expression of the surface molecule CD47 and its binding to SIRP-α on phagocytes efficiently inhibits the uptake of viable cells (57). The induction of CRT surface expression on tumor cells by cytotoxic agents efficiently mediates their uptake by DCs (50). Importantly, CRT-CD91 interaction leads to signaling through NF-κB in DCs and to the release of inflammatory cytokines, in particular TNF, IL-6, IL-1β, and IL-12. This cytokine milieu induced by CRT exposure leads to Th17 priming in an immunosuppressive, TGF-β containing, microenvironment (58).

Adenosine triphophosphate release by dying tumor cells manifests the second hallmark of ICD and is one of the most prominent “find-me” signals for myeloid cells. Upon treatment with selected cytotoxic agents, tumor cells secrete ATP in an autophagy-dependent fashion (53, 59). ATP induces recruitment of myeloid cells into the tumor upon its binding to P2Y2 receptors (55). In the second step, ATP facilitates myeloid cells to differentiate into inflammatory DCs. Furthermore, ATP activates P2RX7 receptors on DCs, which activates the NLRP3 inflammasome leading to IL-1β release. Of note, IL-1β then is required for the priming of CD8^+^ T cells (60). Importantly, priming of T cells appears to occur predominantly in the tumor microenvironment as no significant abrogation in T cell priming is maintained in a mouse model upon surgical removal of draining lymph nodes (55).

High-mobility group box 1 is a chromatin-binding factor found within the nucleus that can be released by injured cells as they undergo primary or secondary necrosis and thereby induces inflammation (61). Treatment of tumor cells by ICD-inducing cytotoxic compounds leads to a post-apoptotic release of HMGB1, which is recognized by TLR4 on DCs. TLR4 controls the tumor antigen processing and is, therefore, indispensable for efficient cross-presentation of tumor cell antigens by DCs (56). In the absence of TLR4 stimulation, phagosomes fuse with lysosomes, which results in degradation of dying cells in the lysosomal compartment, and consequently inefficient antigen presentation (56, 62). In esophageal squamous cell carcinoma, levels of HMGB1 within the tumor microenvironment were significantly upregulated upon preoperative chemoradiotherapy and patients with a high HMGB1 levels showed a better overall survival compared to those with weak HMGB1 expression (63). These findings underline the clinical relevance of HMGB1 expression. However, the inter-patient variability of HMGB1 expression remains poorly understood. HMGB1 can also bind the T cell immunoglobulin- and mucin-domain containing molecule (Tim-3) that is preferentially expressed on tumor-infiltrating DCs (64). Galectin-9 independent ligation of Tim-3 with HMGB1 leads to a negative regulation of nucleic acid-mediated innate immune responses. It is, therefore, reasonable to hypothesize that the balance between a positive signal through TLR4 ligation and a negative signal through Tim-3 ligation might regulate the ICD induced activation of tumor-resident DCs.

While the described mechanisms leading to ICD are able to induce efficient antigen presentation and cytokine secretion, costimulatory molecules might not be upregulated in most of these ICD-inducing chemotherapeutic regiments (50, 55, 56), leaving a gap for more efficient selection of chemotherapeutics or the additional administration of adjuvants.

### Cytotoxic agents targeting tumor-resident immunosuppressive cells

Regulatory T cells interfere with anti-tumor immune responses by several mechanisms [reviewed in Ref. (65)]. For example, Tregs may inhibit DC maturation via CTLA-4/CD80/CD86 interaction and induce expression of the immunosuppressive enzyme indoleamine-2,3-dioxygenase (IDO) (66, 67). Several cytotoxic agents are able to target Tregs and thereby promote adaptive anti-tumor immunity. One of the first drugs reported to interfere with Tregs was cyclophosphamide. At low doses, cyclophosphamide depletes Tregs and inhibits their effector functions and homeostatic proliferation, as demonstrated in mouse models and patients (68–71). Consequently, low-dose cyclophosphamide treatment promotes tumor-specific immune responses when combined with different vaccination strategies, including DC-derived exosomes (DEX) (72) and oncolytic adenovirus (73). Yet, the mechanisms underlying this synergy need to be further elucidated. However, it is reasonable, that in the absence of Tregs, CD4^+^ T cells might activate tumor-resident DCs through CD40L–CD40 interaction, which then can efficiently present tumor antigen and promote T cell activation (74, 75). Additional chemotherapeutic agents targeting Tregs include paclitaxel, which selectively induces apoptosis of Tregs by upregulation of the cell death receptor Fas (76), and low-dose temozolomide, which reduces Treg numbers through poorly understood molecular mechanisms (77, 78).

Myeloid-derived suppressor cells (MDSCs) are a heterogeneous population of immature myeloid cells located in the tumor microenvironment and lymphatic organs. These cells can inhibit innate and adaptive immune responses [reviewed in Ref. (79)]. Several chemotherapeutic drugs can promote anti-tumor immunity by either inducing apoptosis of MDSCs or inducing their differentiation into mature myeloid cells with features of DCs or macrophages. 5-Fluorouracil (5-FU) and gemcitabine substantially reduce the numbers of MDSCs by induction of apoptosis (80, 81). In addition, both 5-FU and gemcitabine induce activation of NLRP3 in dying MDSCs following release of cathepsin B from lysosomes. Active NLRP3 triggers secretion of IL-1β, which may induce IL-17 production by T cells, resulting in priming of Th17 cells, neoangiogenesis, and promotion of tumor growth (82). As 5-FU and gemcitabine, however, do not induce ICD, the measured IL-1β concentrations appear to be low compared to the IL-1β secretion triggered by ICD-inducing chemotherapeutics (80). Importantly, at low concentrations, IL-1β does not support the priming of CD8^+^ T cells and the detrimental effects of IL-1β prevail. Therefore, the therapeutic potential of 5-FU and gemcitabine treatment could be enhanced by co-administration of IL-1β inhibitors. Importantly, 5-FU-induced depletion of MDSCs acts synergistically with Treg depletion induced by low-dose cyclophosphamide treatment, enhancing T cell functions and anti-tumor responses (80). A similar selective depletion of MDSCs and subsequent enhancement of T cell immunity was seen during treatment with doxorubicin or 5-azacytidine (83, 84).

In contrast, non-toxic, low doses of paclitaxel stimulate the differentiation of MDSCs into functional DCs expressing MHCII and costimulatory molecules (85, 86). These functional DCs have lost their suppressive capacity and contribute to the induction of T cell responses. Similarly, docetaxel treatment polarizes MDSCs toward an M1 phenotype with loss of suppressive effects, higher levels of MHCII and CD80 expression, and a shift from IL-10 to IL-12 secretion (87).

## Concluding Remarks

The cytotoxic agents that have been used for several decades for anti-cancer therapy were originally selected for their ability to kill tumor cells. Some, but not all, of these reagents are now known to stimulate anti-tumor immunity, which contributes to their therapeutic effect. A detailed characterization of the immune-stimulatory effects of currently used chemotherapeutic agents should guide the way for rational combinations with immunotherapeutic approaches, which should stimulate anti-tumor immune responses in a synergistic fashion. Cytotoxic agents that directly induce DC maturation or ICD are ideal candidates for combining with inhibitors of immune checkpoints such as PD-1 or CTLA-4, which may result in a long-lasting population of effector memory CD8^+^ T cells (38, 39). In addition, the selective depletion of immune-inhibitory subsets, such as Tregs and MDSCs induced by chemotherapeutic agents, may complement active vaccination strategies and/or checkpoint blockade, by strengthening effector T cell populations (72, 73). The findings discussed here provide the basis for the further development of rational immunotherapeutic protocols in the near future.

## Conflict of Interest Statement

The authors declare that the research was conducted in the absence of any commercial or financial relationships that could be construed as a potential conflict of interest.
